# Evaluation of Detection Methods for Wastewater Surveillance of Antimicrobial-Resistant Bacteria from Healthcare Facilities

**DOI:** 10.3201/eid3213.251490

**Published:** 2026-08

**Authors:** Ean Warren, James VanDerslice, L. Scott Benson, William J. Brazelton, Windy Tanner, Amanda K. Lyons, Florence Whitehill, Angela Coulliette-Salmond, Sydney Fusco, Jennifer Weidhaas

**Affiliations:** University of Utah, Salt Lake City, Utah, USA (E. Warren, J. VanDerslice, L.S. Benson, W.J. Brazelton, W. Tanner, S. Fusco, J. Weidhaas); Centers for Disease Control and Prevention, Atlanta, Georgia, USA (F. Whitehill, A. Coulliette-Salmond); US Public Health Service, Rockville, Maryland, USA (A. Coulliette-Salmond)

**Keywords:** antimicrobial resistance, bacteria, carbapenem resistance, hospital wastewater, sewer biofilm, wastewater-based epidemiology, community surveillance, United States

## Abstract

Carbapenem resistance in bacteria is an urgent public health threat. Wastewater surveillance could support antimicrobial resistance monitoring at long-term care facilities. We assessed feasibility of wastewater sampling at such facilities for carbapenemase genes (*bla*_KPC_, *bla*_VIM_, *bla*_OXA-48-like_, *bla*_NDM_, and *bla*_IMP_) detected by quantitative PCR or GeneXpert Carba-R (Cepheid, https://www.cepheid.com) over 16 months and compared those findings with those for clinical infections. The *bla*_KPC_, *bla*_OXA-48-like_, and *bla*_VIM_ genes were routinely detected in composite wastewater samples, passive samples, and sewer biofilm swab specimens. Wastewater and sewer biofilm resistomes differed, but both contained *bla*_KPC,_ *bla*_VIM,_ and *bla*_IMP_. Wastewater surveillance for carbapenemases shows promise, but few clinical infections during the study period and contributions from sewer biofilms complicate correlations between wastewater measurements and clinical incidence, underscoring the need for further research.

Antimicrobial-resistant infections caused >35,000 deaths in the United States (*1*) and 1,270,000 deaths worldwide (*2*) in 2017; US costs exceeded $13.5 billion (*1*). By 2050, antimicrobial resistance (AMR)–related deaths are projected to rise by 70%, to >208 million (*3*). Among the Centers for Disease Control and Prevention–classified urgent threats are carbapenem-resistant Enterobacterales (CRE), which are responsible for ≈13,100 hospital-associated infections in the United States annually (*1*). Intermediate and long-term acute care facilities are particularly vulnerable to CRE because of high transmission risk and limited treatment options for antimicrobial-resistant infections (*4*).

Routine patient surveillance for infection and colonization by antimicrobial-resistant pathogens is costly (*5*) and invasive (*6*), whereas monitoring hospital wastewater offers a nonnvasive, cost-effective alternative (*7*,*8*) that captures signals of AMR from large patient populations, including colonized persons. Multiple studies have attempted to correlate wastewater AMR genes with infections or hospitalizations in associated communities (*9*–*11*), although few were longitudinal studies (*12*–*14*). One notable study detected a *bla*_NDM_-producing CRE in hospital wastewater that matched a patient isolate (*12*), whereas another found *bla*_CTX-M_ wastewater concentrations were reflective of the patient population (*11*).

Carbapenem-resistant infections are a growing threat in healthcare settings, highlighting the need for practical surveillance methods to detect outbreaks early. Wastewater surveillance (WS) might be such a solution, although sampling techniques might influence wastewater gene concentrations. To address those concerns, we monitored 5 carbapenemase genes of clinical concern (*bla*_KPC_, *bla*_VIM_, *bla*_OXA-48-like_, *bla*_NDM_, and *bla*_IMP_) (*15*) in wastewater from a rehabilitation hospital over 16 months. We collected samples weekly, twice weekly, or monthly using composite, passive, and biofilm-based methods and analyzed them by quantitative PCR (qPCR), metagenomics, and culturing. We evaluated sampling method, hold time, and storage temperature to determine their effects on data reliability; we focused on inexpensive and simple approaches suitable for facilities without nearby diagnostic laboratories. By examining trends in carbapenemase gene abundance, we aimed to establish a baseline of carbapenemase genes for the facility and evaluate whether those trends correlated with clinical infections. 

This study used deidentified patient data correlated with wastewater samples. The University of Utah Institutional Review Board determined the protocol to be exempt under Category 4 of 45 CFR 46.101(b) (protocol no. 00177673; exemption date November 26, 2024). All samples were handled in accordance with biosafety precautions.

## Materials and Methods

### Site Description and Wastewater Parameters

We collected sewer samples from a 5-story rehabilitation hospital with multiple wards in Utah, USA; no other wastewater sources contributed to the flow. With a staff of ≈140, the 75-bed facility provides inpatient and outpatient care (600 outpatient clinical visits per month) and food services. Inpatient care was estimated to contribute 48% of water use, outpatient care 2%, staff 14%, and food services 36% (*16*). Daily visitor count was not known. We confirmed wastewater travel time from various facility floors by flushing dye (Bright Dyes tablet tracer dyes, Kingscote Chemicals, https://www.kingscotechemicals.com) and visually monitoring wastewater in the sewer manhole for arrival of the appropriate dye, as previously reported (*17*).

### Limits of Detection of Antimicrobial-Resistant Genes and Cultures in Composite Wastewater

In brief, we serially diluted bacterial cultures with carbapenemase genes in wastewater (n = 5 for each dilution, n = 3 trials per gene). We then processed samples according to standard methods (Appendix) and determined the viable culture and qPCR gene copies (GC) per milliliter for each dilution.

### Wastewater Methods

#### Sample Collection and Processing

We compared 3 sampling methods (Table 1): composite wastewater collected using a peristaltic sampler (Hach, https://www.hach.com) collected over 24 hours (n = 297), passive absorptive samples (tampons) typically deployed for 24 hours except when field conditions did not allow for 24-hour collection (n = 110), and sewer biofilm swabs (World Bioproducts, https://www.worldbioproducts.com) from the periodically wetted area of the sewer pipe above the flowing wastewater (n = 32) (Appendix). We desorbed bacteria from passive samplers and biofilm swabs by vortex at maximum speed for 10 minutes in 1X phosphorus-buffered saline (PBS) (Thermo Fisher Scientific, https://www.thermofisher.com). We added a spike-in recovery control to all samples (Zymo Research, https://www.zymoresearch.com). We precipitated bacteria by adding polyethylene glycol 6000 and 0.9% of NaCl, shaking (80 RPM), centrifuging (typically 10 minutes at 31,400 × *g*), and resuspending in 1X PBS. We subsampled the resuspended pellet for culturing by using MacConkey I (Avantor, https://www.wvr.com) and CHROMagar KPC agar (DRG International, https://store.drg-international.com), analyzed by using GeneXpert Carba-R (Cepheid, https://www.cepheid.com), and extracted DNA (*18*) for qPCR (*19*–*21*) and metagenomics (Appendix).

**Table 1:** Sample types, frequency of collection, numbers of samples, and handling methods for trend analysis over 16 months in study of detection methods for wastewater surveillance of antimicrobial-resistant bacteria from healthcare facilities*

Sample type	Frequency of collection	No. days of observations	Replicates	Total no. of samples, including replicates†	Handling method‡
Composite	Weekly or twice weekly	61	Primarily laboratory-split triplicates	297	40-mL concentrated for extraction
Sewer biofilm	Weekly or monthly	16	Field duplicates	32	Sponge shaken in sterile 1X PBS for 10 min, supernatant concentrated for extraction
Passive	Weekly or twice weekly	53	Primarily field duplicates	110	Tampon shaken in sterile 1X PBS for 10 min, supernatant concentrated for extraction

*PBS, phosphate-buffered saline. †Total number of samples includes days when more replicates were collected (e.g., 6 replicates rather than the typical 3 replicates). ‡All samples were stored at 4°C until processing, which occurred within 24 hours of collection. All samples were processed by concentration with polyethylene glycol/NaCl precipitation for 2 hours at 4°C, centrifugation at 31,400 × *g*, and then nucleic acids were extracted from the pellet (Appendix).

### Comparison of Sample Volume, Handling, and Storage

We investigated the effects of concentrating 40 mL or 500 mL of sample for gene abundance. The treatments compared 40-mL samples centrifuged at 2 different speeds and times with 500-mL samples (Table 2). We tested those speeds and temperatures with the understanding that not all laboratories have access to high-speed, temperature-controlled centrifuges. After centrifugation, we extracted nucleic acids from the samples (Appendix). We used 1-way analysis of variance (ANOVA) using SigmaStat software version 14.5 (Grafiti, https://grafiti.com) with p<0.05 to assess significant differences among mean carbapenemase GC per milliliter when different water volumes were concentrated. If means were significantly different, we conducted multiple comparison tests using the Holm-Sidak or Dunn method.

**Table 2:** Sample handling methods for comparison of sample volumes, preservation method, hold time, hold temperature, and centrifugation speeds in study of detection methods for wastewater surveillance of antimicrobial-resistant bacteria from healthcare facilities*

Sample type	Comparisons	No. for each treatment	Volume, mL	Hold time, d	Hold temp, °C	Centrifuge settings
G	Sample volumes and centrifugation	4	40	NA	NA	10 min at 31,400 × *g*
G	Sample volumes and centrifugation	2	40	NA	NA	1 h at 3,400 × *g*
G	Sample volumes and centrifugation	4	500	NA	NA	1 h at 3,400 × *g*
C	Control, processed immediately	3	40	NA	NA	10 min at 31,400 × *g*
C	Holding times and temperatures of raw samples	3	40	1, 7, or 29	4, −20, or −70	10 min at 31,400 × *g*
C	Effect of preservatives, hold time and temperatures†	3	40	1, 7, or 29	4, −20, or −70	10 min at 31,400 × *g*
C	PEG concentrated and centrifuged sample with hold	3	40	1	4, −20, or −70	10 min at 31,400 × *g*
C	Nucleic acid stability	3	40	7	−20 or −70	10 min at 31,400 × *g*
C	Nucleic acid stability	6	40	29	−20	10 min at 31,400 × *g*

*C, composite; G, grab sample; NA, not applicable; PEG, polyethylene glycol. †Preservative is 1:1 vol:vol mix of wastewater: sterile 50% glycerol in tris.

We evaluated the effect of sample handling conditions, including hold time, temperature, and processing stage, on gene abundance in 5 treatment types, each with >2 replicates. We concentrated samples using polyethylene glycol centrifugation and resuspension in 1X PBS as described in the previous section. Samples processed immediately served as controls (Table 2). We compared controls with samples held for different days and temperatures before performing concentration and nucleic acid extraction with and without preservation with 1:1 50% glycerol:10 mM 2-amino-2-(hydroxymethyl)propane-1,3-diol (Tris). We used a general linearized model to determine the effect of sample handling and storage conditions on gene abundance (Appendix).

### Wastewater Trends and Alignment with Facility Patient Testing Data

The facility provided monthly average patient census, carbapenem days of therapy, numbers of patients screened for carbapenem-resistant organisms (CRO), numbers of infections with carbapenemase-producing organisms, percentage of patients that were independently performing all steps of toileting and showering, infected patient admission and discharge dates, and building water use. Composite, passive, and sewer biofilm samples were collected weekly during January–December 2023 and then twice monthly during January–April 2024 (Table 1). We determined carbapenemase gene composition in samples by qPCR and Carba-R analysis. We determined total bacteria by quantifying the conserved region of the 16S rRNA gene (Unibac primers) (*25*). We adjusted carbapenemase gene abundance by qPCR for Zymo recovery and log_10_ transformed or normalization by 16S rRNA GC or wastewater flow (Appendix).

We conducted Spearman rank order correlations of carbapenemase gene detections by method and multivariate linear regressions among antibiotic-resistance gene (ARG) abundance in SAS version 9.4 (SAS Institute Inc., https://www.sas.com). We used a nonparametric regression technique to smooth scatter plots of ARG over time using PROC LOESS in SAS with smoothing parameters from 0.1 to 0.25.

## Results

### Limits of Detection of Antimicrobial-Resistant Genes and Cultures in Composite Wastewater

**Figure 1:**
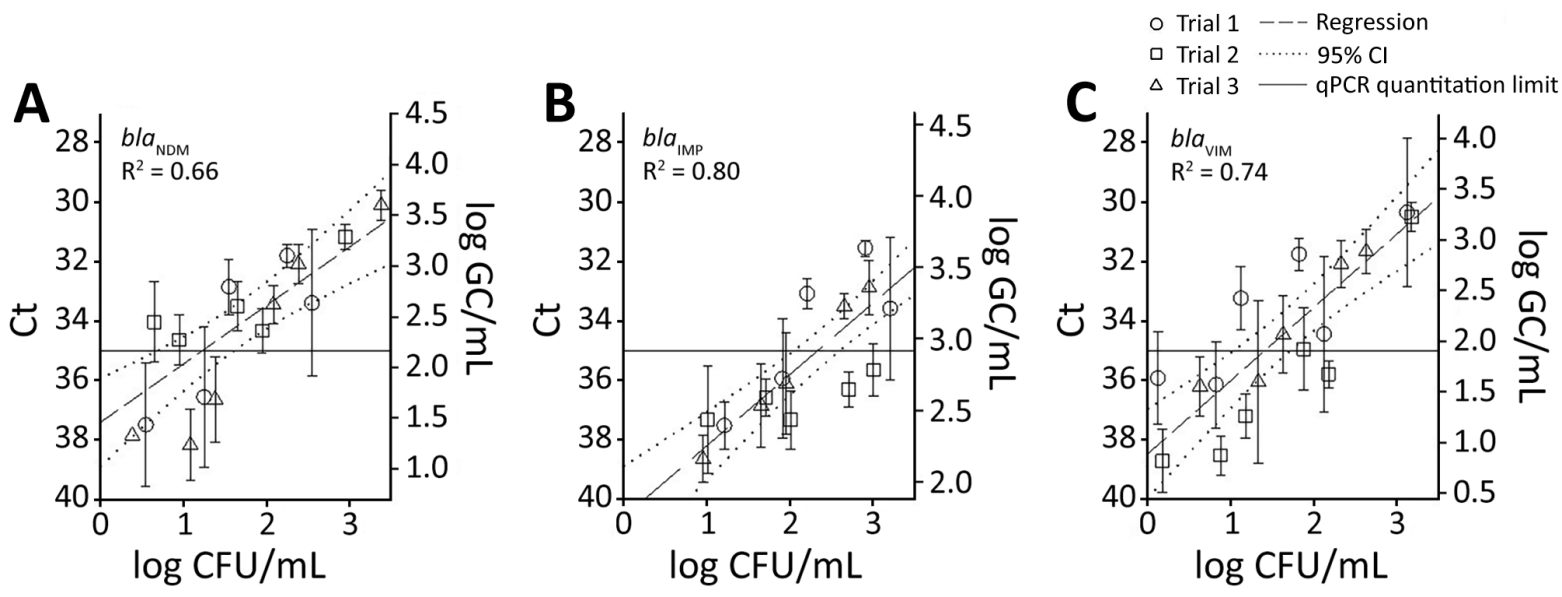
Measured Ct (mean +SD), calculated log GC/mL, and extrapolated log CFU/mL for detection limit studies in study of detection methods for wastewater surveillance of antimicrobial-resistant bacteria from healthcare facilities. A) *bla*_NDM_; B) *bla*_IMP_; C) *bla*_VIM_. Each point represents 5 replicates. Horizontal line represents a Ct cutoff of 35 cycles. All data points were included in the linear regression for each plot; dashed lines represent 95% CIs of the regression. The log CFU/mL was extrapolated from the initial isolate concentration, which was then serially diluted by 6 log into each of 5 replicates. The log GC/mL was estimated from the Ct response from standard curves using either geneblock DNA or antimicrobial-resistant isolates as positive controls. Ct, cycle threshold; GC, gene copies

The detection limits of *bla*_NDM_, *bla*_VIM_, and *bla*_IMP_ in 40 mL of wastewater was 146–5,486 GC/mL with average and SDs of 3 trials (Table 3). We observed variations in cycle threshold (Ct) values for the different dilutions (Figure 1; Appendix). 

**Table 3:** Limits of detection for isolates diluted in wastewater and detected by qPCR and culture-based methods and for qPCR-positive controls diluted in 1X PBS in study of detection methods for wastewater surveillance of antimicrobial-resistant bacteria from healthcare facilities*

Antimicrobial-resistant gene	Observed GC/mL wastewater, mean +SD	Extrapolated culture detection limit, CFU/mL wastewater		Observed qPCR positive control detection limit, GC/μL qPCR reaction§
		Independent estimate, mean +SD†	Combined estimate, limit (95% CI)‡	
*bla*_KPC_	Not estimable	Not estimable		32
*bla*_OXA-48-like_	Not estimable	Not estimable		34
*bla*_VIM_	146 +92¶	38 +41	26 (2.9–865)	241
*bla*_NDM_	284 +154	25 +24	17 (2.2–1,592)	390
*bla*_IMP_	5,486 +3,137	337 +341	216 (4.2–1,5437)	2200

*Not estimable indicates wastewater contained *bla*_KPC_ and *bla*_OXA-48-like_ genes. GC, gene copies; PBS, phosphate-buffered saline; qPCR, quantitative PCR. †The culture detection limit for each of 3 trials was estimated independently based on linear regressions and a cycle threshold (Ct) cutoff of 35; then, the mean and SD of those detection limits was estimated. ‡The detection limit for all 3 trials combined was estimated from the linear regression. §Estimated from qPCR standard curves. ¶Analysis of variance or analysis of variance on ranks was used to determine for each trial the mean Ct value for the lowest dilution that was above a Ct of 35 and at a significantly different concentration than the next lowest dilution. The qPCR standard curve calculation was then used for this Ct to estimate the GC/mL. Mean and SD GC/mL for the 3 trials was then estimated.

### Wastewater Methods

#### Comparison of Composite, Passive and Biofilm Sampling Methods

**Figure 2:**
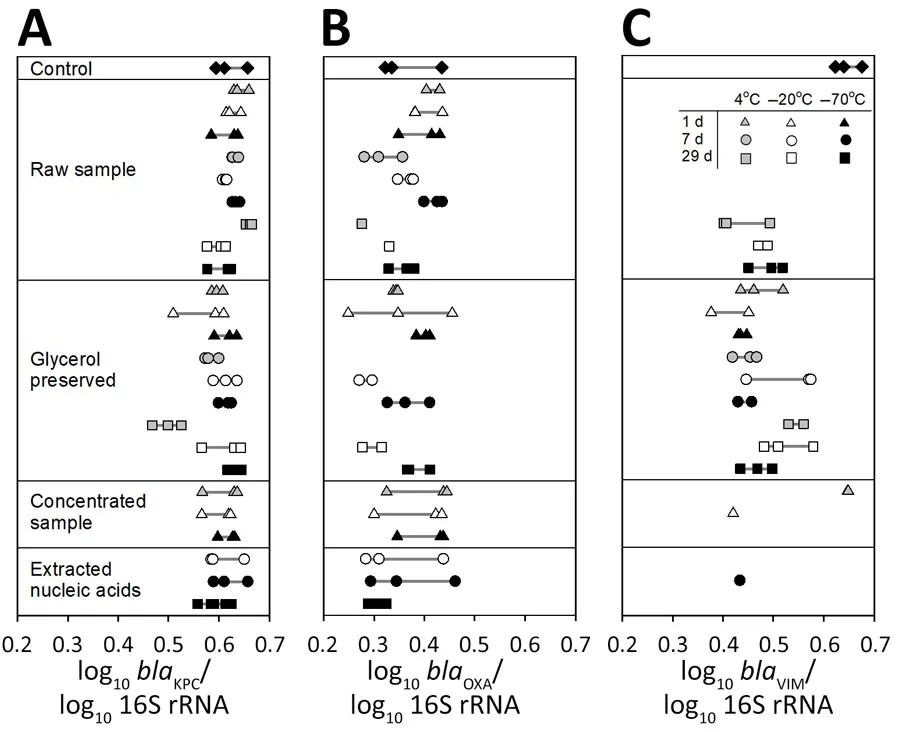
Holding time and preservation temperature comparison for log_10_ *bla*_KPC_ (A), *bla*_OXA-48-like_ (B), and *bla*_VIM_ (C) measured by quantitative PCR in triplicate in study of detection methods for wastewater surveillance of antimicrobial-resistant bacteria from healthcare facilities. Triangles indicate 1 day holding time, circles indicate 7 days, and squares indicate 29 days. Gray symbols indicate 4°C. white symbols indicate –20°C, and black symbols indicate –70°C. All gene copies are normalized to log_10_ 16S rRNA gene. Horizontal gray lines show the range of values for each treatment. Control sample DNA was extracted immediately upon receipt in laboratory. Raw sample represents processed raw wastewater. Glycerol preserved are unprocessed samples preserved with 50% glycerol with Tris. Concentrated samples were polyethylene glycol precipitated for 2 hours and centrifuged, and the resulting pellet was resuspended and held at the listed temperature.

The *bla*_KPC_, *bla*_VIM_, and *bla*_OXA-48-like_ GC normalized by water usage showed a strong correlation among composite wastewater and passive samples (Spearman ρ<0.05) (Table 4; Appendix Figure 1, whereas *bla*_NDM_ and *bla*_IMP_ were not correlated. In biofilm samples, *bla*_OXA_ was correlated with passive samples and with *bla*_KPC_. We observed inverse correlations between *bla*_KPC_ in biofilms and *bla*_VIM_ in passive samplers but only in 7 samples, thus decreasing the power of the analysis. Regardless of method, fewer gene concentrations were correlated when the data were not water usage–normalized (Appendix Table 5, Figure 2). When normalized to facility water usage, sewer biofilm qPCR concentrations correlated with composite wastewater samples. Deployment of passive samplers for >24 hours (n = 29) was shown to increase only total bacteria (i.e., conserved region of 16S rRNA gene) and *bla*_KPC_ (p<0.05) (i.e., 0.8-day to 7-day deployment with 2.3 +1.5 day, [average + SD]), but R^2^ values were all <0.15 (Appendix Figure 3).

**Table 4:** Spearman rank order correlations among building water usage-normalized *bla*_KPC_, *bla*_OXA-48-like_, and *bla*_VIM_ log gene concentrations for composite, passive, and sewer biofilm sample collection methods in study of detection methods for wastewater surveillance of antimicrobial-resistant bacteria from healthcare facilities*

Gene	Composite				Passive				Biofilm		
	KPC	OXA	VIM		KPC	OXA	VIM		KPC	OXA	VIM
Composite											
KPC	–	ρ = 0.66, p<0.001, n = 58	ρ = 0.56, p = 0.008, n = 21		ρ = 0.40, p = 0.003, n = 51	ρ = 0.41, p = 0.003, n = 50	ρ = 0.68, p = 0.005, n = 15		NS	NS	NS
OXA		–	ρ = 0.64, p = 0.002, n = 21		NS	ρ = 0.4,1 p = 0.003, n = 50	ρ = 0.70, p = 0.004, n = 15		NS	NS	NS
VIM			–		NS	NS	ρ = 0.78, p = 0.005, n = 10		NS	NS	NS
Passive											
KPC					–	ρ = 0.60, p<0.001, n = 51	NS		NS	NS	NS
OXA						–	NS		NS	ρ = 0.67, p = 0.008, n = 14	NS
VIM							–		ρ = −0.821, p = 0.01, n = 7	NS	NS
Biofilm											
KPC									–	ρ = 0.76 p<0.001, n = 15	NS
OXA										–	NS
VIM											–

*Daily values were calculated for composite samples as log(GC/mL)/(monthly average gal × 10^3^/d), for passive samplers as log(GC/g)/(monthly average gal × 10^3^/d), and for biofilm samples as log(GC/cm^2^)/(monthly average gal × 10^3^/d); nonnormalized data correlations are shown in Appendix Table 5. *bla*_NDM_ and *bla*_IMP_ are not included because they were not correlated or only correlated in <5 samples. B, sewer biofilm; C, composite; CKPC, *bla*_KPC_; NS, not significant at p<0.05; OXA, *bla*_OXA-48-like_; P, passive; VIM, *bla*_VIM_.

We correlated CHROMagar KPC and MacConkey I culture counts in passive or composite samples for *E. coli*, *Pseudomonas* spp., and *Acinetobacter* spp. (Appendix Tables 6, 7). Metagenome sequencing revealed a wide range of ARGs (Appendix Figure 4), including detection of *bla* genes in all sample types (Appendix Figure 5). All target *bla* genes were detected in the metagenomes of >1 sample (Appendix Figure 4). Passive samples had more *bla*_KPC_ (Appendix Figure 5) than the biofilm and grab metagenomes, which had very low abundances (1–5 transcripts/million [TPM]). The *bla*_VIM_ gene was most abundant (14 TPM) in the biofilm metagenome. Neither *bla*_NDM_ nor *bla*_OXA-48-like_ genes were detected in any metagenomes, despite detection by qPCR. We examined the relationship between metagenomic sequence coverage and qPCR abundance (Appendix Figure 6) and found a significant correlation for *bla*_KPC_ (R^2^ = 0.66). In contrast, *bla*_VIM_ was only detected in 1 of 3 metagenomes, where the qPCR abundance was 4 log_10_ GC. The *bla*_OXA-48-like_ abundance was 2 to 6 log_10_ GC and the *bla*_NDM_ abundance was <5 log_10_ GC, but they were not detected in metagenomes. Therefore, the absence of *bla*_OXA-48-like_ and *bla*_NDM_ genes in the metagenomes is surprising but reasonable because of the limits of detection for metagenomic sequencing.

**Table 6:** Concentrations of carbapenemase genes in composite wastewater by month and frequency of carbapenemase gene detection over 16 months in study of detection methods for wastewater surveillance of antimicrobial-resistant bacteria from healthcare facilities*

Month	No. sampling dates	log GC/mL, mean +SD (no. [%] detected)†				
		*bla*_KPC_	*bla*_OXA-48-like_	*bla*_VIM_	*bla*_NDM_	*bla*_IMP_
2023 Jan	1	7.8 (1 [100])	6.3 (1 [100])	8.7 (1 [100])	ND	ND
2023 Feb	2	7.1 + 0.1 (2 [100])	6.2 + 0.4 (2 [100])	7.8 + 0.3 (2 [100])	ND	ND
2023 Mar	4	7.3 + 0.3 (4 [100])	5.8 + 0.2 (4 [100])	7.3 (1 [25])	ND	ND
2023 Apr	3	6.5 + 0.6 (3 [100])	4.9 + 0.5 (3 [100])	ND	ND	ND
2023 May	8	6.9 + 0.5 (8 [100])	5.8 + 0.8 (8 [100])	6.1 + 0.6 (2 [25])	ND	ND
2023 Jun	9	6.5 + 0.4 (9 [100])	5.4 + 0.5 (9 [100])	5.4 + 0.5 (2 [22])	ND	ND
2023 Jul	7	6.3 + 0.6 (7 [100])	5.8 + 0.6 (6 [86])	4.2 (1 [14])	ND	ND
2023 Aug	6	5.2 + 0.6 (6 [100])	5.2 + 0.7 (6 [100])	5.5 + 0.5 (2 [33])	ND	ND
2023 Sep	4	6.1 + 0.8 (4 [100])	5.0 + 0.5 (4 [100])	5.2 (1 [25])	ND	ND
2023 Oct	3	6.3 + 0.5 (3 [100])	5.4 + 0.1 (3 [100])	5.3 + 0.2 (2 [67])	4.6 (1 [33])	ND
2023 Nov	4	6.1 + 0.4 (4 [100])	4.8 + 0.3 (4 [100])	4.8 + 0.2 (3 [75])	5.7 (1 [25])	ND
2023 Dec	2	5.5 + 0.1 (2 [100])	4.4 + 0.2 (2 [100])	4.8 (1 [50])	ND	ND
2024 Jan	2	5.6 + 0.2 (2 [100])	4.8 + 0.5 (2 [100])	ND	ND	ND
2024 Feb	2	6.1 + 0.3 (2 [100])	5.7 + 0.3 (2 [100])	5.6 + 0.2 (2 [100])	5.1 + 0.6 (2 [100])	ND
2024 Mar	2	5.8 + 0.9 (2 [100])	5.1 + 0.6 (2 [100])	4.8 (1 [50])	4.6 + 0.6 (2 [100])	6.5 (1 [50])
2024 Apr	2	5.9 + 0.5 (2 [100])	5.0 + 0.4 (2 [100])	ND	5.3 + 0.4 (2 [100])	6.6 (1 [50])
Total (% positive)		61 (100)	60 (98)	21 (34)	8 (13)	2 (3)

*GC, gene copies; ND, not detected. †Concentrations without SD indicate carbapenemase genes were detected in only 1 sample among multiple sampling dates in a month or only 1 sampling date in a month.

**Table 7:** Concentrations of carbapenemase genes collected with passive samplers by month and frequency of carbapenemase gene detection over 16 months in study of detection methods for wastewater surveillance of antimicrobial-resistant bacteria from healthcare facilities*

Month, no. samples	log GC/mL, mean +SD (no. [%] detected)†				
	*bla*_KPC_	*bla*_OXA-48-like_	*bla*_VIM_	*bla*_NDM_	*bla*_IMP_
2023 Jan, n = 1	8.03 (1 [100])	6.8 (1 [100])	7.9 (1 [100])	ND	ND
2023 Feb, n = 2	7.5 + 0.1 (2 [100])	5.9 + 0.1 (2 [100])	7.9 + 0.2 (2 [100])	ND	ND
2023 Mar	Not tested	Not tested	Not tested	Not tested	Not tested
2023 Apr, n = 1	9.0 (1 [100])	6.2 (1 [100])	ND	ND	ND
2023 May, n = 8	8.5 + 0.6 (8 [100])	7.0 + 0.7 (8 [100])	ND	ND	ND
2023 Jun, n = 9	8.3 + 0.5 (9 [100])	6.9 + 0.4 (9 [100])	6.8 (1 [11])	ND	ND
2023 Jul, n = 7	8.3 + 0.3 (6 [86])	6.5 + 0.2 (6 [86])	ND	ND	ND
2023 Aug, n = 7	7.9 + 0.3 (7 [100])	6.4 + 0.1 (6 [86])	ND	ND	ND
2023 Sep, n = 3	8.0 + 0.5 (3 [100])	6.4 + 0.4 (3 [100])	ND	ND	ND
2023 Oct, n = 3	7.9 + 0.2 (3 [100])	6.1 + 0.1 (3 [100])	6.4 (1 [33])	5.4 (1 [33])	ND
2023 Nov, n = 4	8.1 + 0.4 (4 [100])	6.1 + 0.2 (4 [100])	6.0 + 0.2 (3 [75])	6.0 (1 [25])	ND
2023 Dec, n = 2	7.8 + 0.001 (2 [100])	6.3 + 0.2 (2 [100])	6.4 + 0.1 (2 [100])	ND	ND
2024 Jan, n = 2	7.9 + 0.03 (2 [100])	6.5 + 0.1 (2 [100])	6.3 + 0.1 (2 [100])	ND	ND
2024 Feb, n = 2	8.3 + 0.7 (2 [100])	6.8 + 0.5 (2 [100])	6.4 + 0.2 (2 [100])	5.1 (1 [50])	7.9 + 0.3 (2 [100])
2024 Mar, n = 2	8.2 + 0.7 (2 [100])	7.0 + 0.9 (2 [100])	6.2 (1 [50])	5.4 (1 [50])	7.8 (1 [50])
2024 Apr, n = 2	8.0 + 0.6 (2 [100])	6.7 + 0.2 (2 [100])	6.2 + 0.5 (2 [100])	6.1 (1 [50])	7.6 + 0.2 (2 [100])
Total (% positive), n = 55	54 (98)	53 (96)	17 (31)	5 (9)	5 (9)

*GC, gene copies; ND, not detected. †Concentrations without SD indicate carbapenemase genes were detected in only 1 sample among multiple sampling dates in a month or only 1 sampling date in a month.

We sequenced and classified 7 isolates from composite wastewater in January 2023 as *Citrobacter*, *E. coli* (1 isolate; serotype O8 + H19), and *Providencia* (1 isolate). Of 12 isolates sequenced and classified in October 2023, 6 of 12 were also *Citrobacter*, 3 were *Comamonas*, and 3 were *Aeromonas*. The *Aeromonas* and *Citrobacter* isolates had similar ARGs (Appendix Figure 7), including *bla*_KPC-2_, *bla*_KPC-3_, *bla*_OXA-2_, *bla*_TEM-1_, and *bla*_SHV_. The *E. coli* genome includes *bla*_CTX-M-15_, *bla*_EC_, and *bla*_TEM-1_.

#### Comparison of Carbapenemase Gene Detection Methods

Among all samples and collection methods, there was close agreement between the Carba-R assay and qPCR detections (Appendix Tables 10–12); 551 (85.4%) of 645 replicate carbapenemase gene assay results were either both present or both absent. The *bla*_VIM_ gene was more frequently detected by Carba-R assay than by qPCR; 76 (58.9%) of 129 samples were positive by Carba-R but negative by qPCR. The *bla*_IMP_ gene was not detected by Carba-R, whereas 7% (5/69) of samples were qPCR positive.

#### Comparison of Composite Wastewater Sample Volume, Handling, and Storage

We conducted studies to evaluate the effect of wastewater sample volume, sample concentration method, and sample handling methods on ARG abundance. In the first study of wastewater sampling volumes, we found no significant difference (p>0.05 by Dunn method) in gene abundance among 2 40-mL samples centrifuged at different speeds (31,400 × *g* or 3,400 × *g*) and times (10 or 60 minutes); therefore, we combined those data for further analysis. Overall, 40-mL samples had a significantly higher mean abundance than the 500-mL samples for *bla*_VIM_ (p<0.05 by Holm-Sidak method), 16S rRNA gene (p = 0.02 by ANOVA on Ranks), and *bla*_OXA-48-like_ (p = 0.01 by ANOVA on Ranks), but not for *bla*_KPC_ (p = 0.1 by ANOVA) (Appendix Figure 8). Therefore, we used 40-mL samples with centrifugation at 31,400 × *g* for 10 minutes.

We compared sample holding times (1, 7, and 29 days), holding methods (concentrated before storage or not), and preservation methods (raw sample or glycerol preserved) with samples extracted and analyzed immediately to determine how sample handling influenced ARG abundance (Table 5, Figure 2). Sample preservation method (Table 5) generated the most variation in gene abundance. Specifically, the sample preservation method accounted for 24%–42% of the variation in GC per milliliter. The highest recovery was observed in concentrated samples (57%–121% average recovery of ARG among all temperatures and times) or extracted nucleic acid samples (59%–94% recovery). Sample hold time before handling accounted for 25% to 38% of variability. Hold time significantly influenced ARG abundance (Table 5); shorter hold times are recommended.

**Table 5:** Effects of sample handling and storage conditions on gene abundance determined by qPCR in study of detection methods for wastewater surveillance of antimicrobial-resistant bacteria from healthcare facilities*

Factor	16S rRNA gene	*bla*_KPC_	*bla*_VIM_	*bla*_OXA-48-like_	*bla*_KPC_/16S rRNA†	*bla*_VIM_/16S rRNA†
Overall model F (p value)	1.98 (0.02)	2.23 (0.008)	6.33 (<0.001)	NS	3.99 (<0.01)	9.67 (<0.001)
Source of variation						
Hold time‡	38 (0.002)	25 (0.01)	30 (<0.001)	NA§	NS	31 (<0.001)
Hold temp¶	NS	NS	16 (0.01)	NA	NS	10 (0.008)
Hold method	41 (<0.001)	42 (<0.001)	30.1 (<0.001)	NA	27 (<0.001)	24 (<0.001)
Time × temp	NS	NS	12.5 (0.008)	NA	NS	11 (0.002)
Temp × method	NS	NS	NS	NA	NS	15 (0.007)
Time × method	NS	NS	9 (0.04)	NA	22 (<0.001)	9 (0.006)
Time × temp × method	NS	15 (0.05)	Not estimable**	NA	27 (<0.001)	Not estimable**

*Source of variation is percent of total variation (p value) of type 1 error for the general linearized model. NA, not applicable; NS, not significant; qPCR, quantitative PCR. †Calculation based on log_10_ ARG/mL divided by log_10_ 16S rRNA gene/mL. ‡Hold times were 1, 7, or 29 days. §The overall model was not significant for this gene. ¶Temperature was 4°C, −20°C, or −70°C. #Hold methods: extracted immediately versus raw samples held at temperature, glycerol preserved unprocessed samples, polyethylene glycol concentrated and resuspended samples, or nucleic acids extracted and stored. **These values were not estimable because of limited degrees of freedom.

### Wastewater Carbapenemase Gene Trends and Alignment with Facility Clinical Infections

During the 16-month study, only 5 CRO clinical infections were identified in 4 patients within the facility. Infections 1 and 2 were identified as *Klebsiella (Enterobacter) aerogenes* and *Enterobacter cloacae*, isolated from urine specimens collected 9 days apart in April 2023 from the same patient; both were negative for carbapenemase genes. Infection 3 was a carbapenem-resistant *Pseudomonas aeruginosa* (CRPA) from a wound in April 2023, which was not tested for carbapenemase genes. Infection 4 was a case of CRPA from a urine specimen in December 2023, also not tested for carbapenemase genes. Infection 5 was determined to be *Escherichia coli* and *Citrobacter freundii* and came from a perianal screen in March 2024; it was not tested for carbapenemase genes. Of note, the patient with CRE identified in urine specimens in April 2023 used ostomy bags, which might not have been disposed of in the toilet. All other patients were recorded as toileting by nurses. Carbapenem antibiotic use in the facility was documented (Appendix Table 13).

Over 16 months (Tables 6, 7), *bla*_KPC_ and *bla*_OXA-48-like_ were detected in >98% of composite and passive samples, but *bla*_VIM_, *bla*_NDM_, and *bla*_IMP_ were detected less frequently (35% in composite and 31% in passive for *bla*_VIM_, 10% in composite and 9% in passive for *bla*_NDM_, 3% in composite and 9% in passive for *bla*_IMP_). All sewer biofilm samples contained *bla*_KPC_ and *bla*_OXA-48-like_, but only 24% of samples contained *bla*_NDM_ and *bla*_VIM_, and 18% contained *bla*_IMP_ (Table 8).

**Table 8:** Concentrations of carbapenemase genes observed in sewer biofilms by month and frequency of carbapenemase gene detection over 16 months in study of detection methods for wastewater surveillance of antimicrobial-resistant bacteria from healthcare facilities*

Month, no. samples	log GC/cm2, mean +SD (no. [%] detected)†				
	*bla*_KPC_	*bla*_OXA-48-like_	*bla*_VIM_	*bla*_NDM_	*bla*_IMP_
2023 Jan, n = 1	4.6 (1 [100])	3.9 (1 [100])	6.9 (1 [100])	ND	ND
2023 Feb, n = 1	4.4 (1 [100])	3.9 (1 [100])	6.0 (1 [100])	ND	ND
2023 Mar, n = 1	5.6 (1 [100])	3.5 (1 [100])	ND	ND	ND
2023 Apr	Not tested	Not tested	Not tested	Not tested	Not tested
2023 May, n = 1	6.9 (1 [100])	5.7 (1 [100])	ND	ND	ND
2023 Jun, n = 1	6.0 (1 [100])	5.0 (1 [100])	ND	ND	ND
2023 Jul	Not tested	Not tested	Not tested	Not tested	Not tested
2023 Aug, n = 1	4.5 (1 [100])	4.0 (1 [100])	ND	ND	ND
2023 Sep, n = 1	6.3 (1 [100])	5.6 (1 [100])	ND	ND	ND
2023 Oct, n = 1	5.4 (1 [100])	4.5 (1 [100])	ND	ND	ND
2023 Nov, n = 3	5.6 + 0.8 (3 [100])	4.2 + 1.1 (3 [100])	ND	ND	ND
2023 Dec, n = 1	4.8 (1 [100])	4.1 (1 [100])	5.0 (1 [100])	ND	ND
2024 Jan, n = 1	5.0 (1 [100])	4.1 (1 [100])	ND	ND	ND
2024 Feb, n = 1	5.3 (1 [100])	5.5 (1 [100])	ND	6.8 (1 [100])	ND
2024 Mar, n = 1	5.1 (1 [100])	4.8 (1 [100])	ND	4.5 (1 [100])	6.5 (1 [100])
2024 Apr, n = 2	4.3 + 0.7 (2 [100])	4.5 + 0.2 (2 [100])	4.2 (1 [50])	5.0 + 0.1 (2 [100])	6.3 + 0.2 (2 [100])
Total (% positive), n = 17	17 (100)	17 (100)	4 (24)	4 (24)	3 (18)

*GC, gene copies; ND, not detected. †Concentrations without SD indicate carbapenemase genes were detected in only 1 sample among multiple sampling dates in a month or only 1 sampling date in a month.

**Figure 3:**
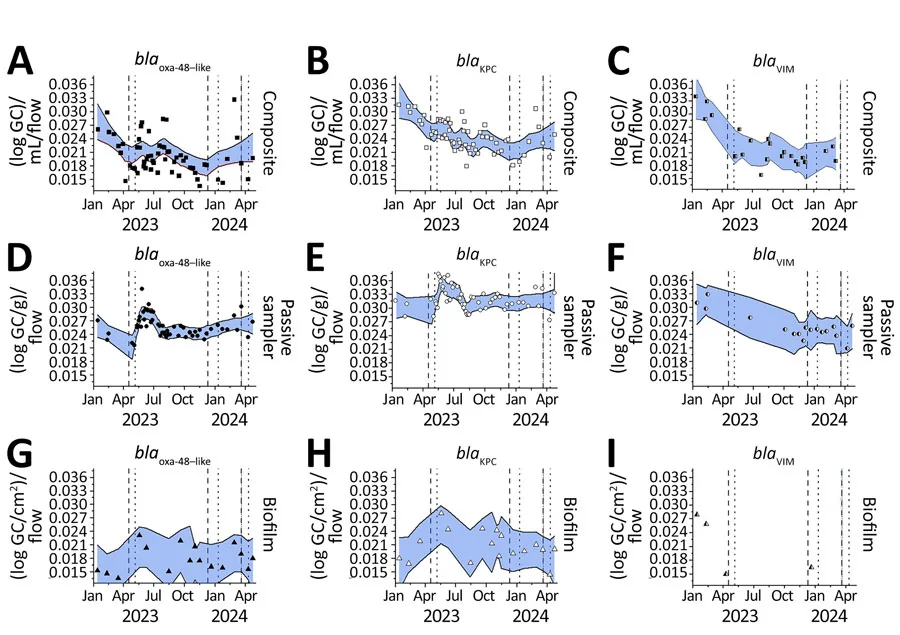
GC of *bla*_OXA-48-like_, *bla*_KPC_, and *bla*_VIM_ in wastewater collected by autosamplers, passive samplers, or swabs of sewer biofilm in study of detection methods for wastewater surveillance of antimicrobial-resistant bacteria from healthcare facilities, January 2023–April 2024. A–C) Composite samples for *bla*_OXA-48-like_, *bla*_KPC_, and *bla*_VIM_. D–F) Passive sampler results for *bla*_OXA-48-like_, *bla*_KPC_, and *bla*_VIM_. G–I) Biofilm samples for *bla*_OXA-48-like_, *bla*_KPC_, and *bla*_VIM_. Values are normalized by recovery control and water usage. Nonparametric regression smoothing factors range from 0.1 to 0.25. Dashed lines (admit date) and dotted lines (discharge date) indicate periods when patients infected with carbapenem-resistant Enterobacterales (CRE) or carbapenem-resistant *Pseudomonas aeruginosa* were in the facility. Infections consisted of *Klebsiella aerogenes*, *Enterobacter cloacae*, and *Pseudomonas aeruginosa* in period 1 (April 2023); *P. aeruginosa* in period 2 (December 2023); and *Escherichia coli* and *Citrobacter freundii* in period 3 (March 2024). The CRE infections in April 2023 were not carbapenemase-producing organisms, based on PCR. The CRE infection in March 2024 was not tested for carbapenemase genes by PCR. None of the carbapenem-resistant *Pseudomonas aeruginosa* were tested for carbapenemase genes.

Abundance of *bla*_KPC_ and *bla*_OXA-48-like_, normalized for facility water usage, increased after April 2023 when CRE- and CRPA-infected patients were present (Figure 3). A general increase in *bla*_KPC_ and *bla*_OXA-48-like_ abundance was also observed in the last 4 months of the study period (Figure 3, panels A, B, D, and E), although that increase was not attributable to a patient infection. The *bla*_VIM_ abundance generally decreased over the study period for flow-normalized and nonnormalized data (Figure 3, panels G, H, I; Appendix Figure 9). The low incidence of CRO infections (5 over 16 months) and lack of confirmed carbapenemase-producing CRE infections precluded modeling of relationships between wastewater carbapenemase gene abundances and clinical infection occurrence.

## Discussion

This study standardized methods for carbapenemase gene detection in hospital wastewater. Sample handling conditions significantly affected gene abundance measurements. Gene recovery was highest when 40 mL of sample was processed using a higher centrifuge speed, compared with 500 mL processed at lower centrifugation speeds. Recovery decreased with prolonged storage or higher temperature unless samples were preserved with glycerol. Those findings emphasize the importance of standardized, practical protocols (*22*). Our results demonstrate that passive sampling is as effective as composite sampling, as suggested by others (*23*), and that storing raw samples at 4°C up to 29 days was comparable to immediate processing. That finding is especially relevant for resource-limited settings, because those facilities can collect and store a passive sample before processing in lieu of composite samples processed the next day.

Over 16 months, 2 carbapenemase genes, *bla*_KPC_ and *bla*_OXA-48-like_, were consistently detected by qPCR and Cepheid Carba-R assays in each of the 3 collection methods. Among sampling strategies, the composite and passive sampling outperformed biofilm sampling in terms of gene richness and antimicrobial-resistant gene detection frequency. Passive samples also yielded higher concentrations of carbapenemase genes. The sewer biofilm gene concentrations when normalized to building water usage was correlated with wastewater concentrations, suggesting the sewer biofilm might influence the wastewater carbapenemase gene abundance, but additional studies are needed to elucidate that relationship. Differences in gene abundance between sewer biofilms and composite samples might reflect shedding from colonized patients, environmental persistence, or microbial community dynamics.

Temporal trends of gene abundance in wastewater were not correlated with patient infections because of the limited clinical infections during the study. Longer-term sampling is required to evaluate the utility of WS as a complement to hospital AMR surveillance methods. Two critical points require further consideration: first, wastewater results should be interpreted considering reservoirs for antimicrobial-resistant bacteria, such as sinks and sewer biofilms, which were found to harbor carbapenemase genes (Appendix Figure 5) and CRE as reported elsewhere (*24*,*25*); and second, CRE or carbapenemase gene sources in addition to infected patients such as colonized patients, hospital staff, outpatients, and visitors should be considered. Additional research is needed to investigate the contribution of sewer biofilms to wastewater from hospitals, because those biofilm signals might confound temporal gene signals. Identifying the nonpatient contribution of CRE or carbapenemase genes to wastewater will also be critical for successful surveillance efforts.

Because of the limited clinical infections during the study period, it remains an open question whether WS might enhance outbreak detection, support infection control, and aid antibiotic stewardship, and additional study is needed. Further, the contribution of sewer biofilms to wastewater signals needs investigation. In conclusion, wastewater sampling, particularly composite and passive methods combined with sensitive molecular detection, might be a practical, noninvasive approach for monitoring carbapenemase genes in healthcare facilities, as demonstrated by other reports (*26*,*27*). Passive sampling might be suitable in low-resource settings, situations with space constraints, or situations in which sewer access is limited. 

This article was preprinted at https://www.biorxiv.org/content/10.64898/2025.12.14.694051v1.

Appendix Additional information about detection methods for wastewater surveillance of antimicrobial-resistant bacteria from healthcare facilities.
